# Retrieval-augmented generation elevates local LLM quality in radiology contrast media consultation

**DOI:** 10.1038/s41746-025-01802-z

**Published:** 2025-07-02

**Authors:** Akihiko Wada, Yuya Tanaka, Mitsuo Nishizawa, Akira Yamamoto, Toshiaki Akashi, Akifumi Hagiwara, Yayoi Hayakawa, Junko Kikuta, Keigo Shimoji, Katsuhiro Sano, Koji Kamagata, Atsushi Nakanishi, Shigeki Aoki

**Affiliations:** 1 Department of Radiology, Juntendo University Graduate School of Medicine, 2-1-1 Hongo, Bunkyo-ku, Tokyo 113-8421 Japan; 2 Department of Radiology, The University of Tokyo School of Medicine, 7-3-1 Hongo, Bunkyo-ku, Tokyo 113-8655 Japan; 3 Department of Radiology, Juntendo University Urayasu Hospital, 2-1-1 Tomioka, Urayasu, Chiba 279-0021 Japan; 4 Faculty of Health Data Science, Juntendo University Graduate School of Medicine, 6-8-1 Hinode, Urayasu, Chiba 279-0013 Japan

**Keywords:** Medical imaging, Health care

## Abstract

Large language models (LLMs) demonstrate significant potential in healthcare applications, but clinical deployment is limited by privacy concerns and insufficient medical domain training. This study investigated whether retrieval-augmented generation (RAG) can improve locally deployable LLM for radiology contrast media consultation. In 100 synthetic iodinated contrast media consultations we compared Llama 3.2-11B (baseline and RAG) with three cloud-based models—GPT-4o mini, Gemini 2.0 Flash and Claude 3.5 Haiku. A blinded radiologist ranked the five replies per case, and three LLM-based judges scored accuracy, safety, structure, tone, applicability and latency. Under controlled conditions, RAG eliminated hallucinations (0% vs 8%; χ²₍Yates₎ = 6.38, p = 0.012) and improved mean rank by 1.3 (Z = –4.82, p < 0.001), though performance gaps with cloud models persist. The RAG-enhanced model remained faster (2.6 s vs 4.9–7.3 s) while the LLM-based judges preferred it over GPT-4o mini, though the radiologist ranked GPT-4o mini higher. RAG thus provides meaningful improvements for local clinical LLMs while maintaining the privacy benefits of on-premise deployment.

## Introduction

Large language models (LLMs) have rapidly expanded across domains, demonstrating significant potential to enhance human capabilities and workflow efficiency in specialized fields that require domain-specific knowledge. In medicine, LLMs are promising to summarize medical literature, support clinical reasoning, and extract key information from medical records^1,2^. In radiology, recent studies highlight LLMs’ potential to improve report quality, workflow efficiency, and diagnostic accuracy^3,4^.

Despite this potential, healthcare implementation of LLMs faces unique challenges centered on a fundamental dilemma: balancing patient data confidentiality with advanced AI capabilities. Cloud-based LLMs offer superior reasoning abilities through large-scale training data but require transmitting sensitive medical information to external servers, raising substantial concerns under regulations like HIPAA and GDPR^5^.

Conversely, locally deployed LLMs ensure data privacy but typically demonstrate inferior performance due to constraints in model size and computational resources^6^. This performance gap is particularly pronounced in specialized medical domains where domain-specific knowledge and nuanced understanding of clinical guidelines are essential.

Retrieval-augmented generation (RAG) has emerged as a promising approach to address this trade-off. By integrating external domain-specific knowledge into the response generation process, RAG enables LLMs to leverage specialized information beyond their training data, reduce hallucinations through authoritative source grounding, and dynamically update knowledge without additional model fine-tuning^7^. RAG combines high accuracy with robust data privacy when implemented within a locally deployed environment, potentially offering a solution to the privacy-performance dilemma in healthcare AI.

While RAG’s effectiveness has been demonstrated in general knowledge retrieval tasks, its utility in highly specialized medical fields, particularly radiology, remains underexplored. Previous studies have identified knowledge base quality and retrieval efficiency as key determinants of RAG performance in medical applications^8,9^, but few have evaluated its application in time-sensitive clinical decision support scenarios^10^.

To systematically assess locally deployed RAG-enhanced LLMs in a specialized medical context, this study focuses on iodinated contrast media (ICM) consultations—a crucial radiological task requiring specialized knowledge and real-time decision-making. CT examinations have become fundamental to modern diagnostic imaging, with annual scan volumes in the United States increasing from approximately 3 million in 1980 to 91 million in 2019^11^. Notably, 40-48% of all CT scans involve ICM to enhance visualization of vascular and organ structures, making appropriate contrast administration essential to radiological practice.

Recent challenges have amplified the importance of precise ICM consultation. The COVID-19 pandemic caused severe global contrast agent shortages, necessitating careful assessment of contrast-enhanced scan appropriateness^12^. Additionally, ICM-related adverse events range from mild allergic reactions to severe nephrotoxicity, making risk stratification crucial for patient safety. Research indicates that 7.5% of ICM-related complications could be prevented through appropriate consultation^13^, while communication errors in contrast media ordering account for 13.9% of radiology workflow inefficiencies^14^.

Effective ICM consultation requires specialized radiological knowledge, clinical experience, and dynamic risk assessment capabilities. LLMs could potentially assist radiologists by automating risk assessment and protocol selection, thereby reducing workload and enhancing decision-making. The integration of facility-specific protocols and recently updated guidelines through RAG could be particularly valuable, as contrast media administration often involves institution-specific rules that cannot be captured in general model training.

This study aims to evaluate the performance of RAG-enhanced lightweight local LLMs in ICM consultation compared to leading cloud-based models. We developed and assessed a RAG-implemented locally deployable model (Llama 3.2 11B) against three cloud-based LLMs (GPT-4o mini, Gemini 2.0 Flash, Claude 3.5 Haiku) using 100 simulated clinical scenarios requiring ICM risk assessment and protocol selection. Both radiologists and automated LLM evaluators evaluated performance through a comprehensive assessment, examining clinical accuracy, safety, response structure, communication quality, practical applicability, and response time.

By quantifying performance differences between cloud-based and locally deployed models and assessing whether RAG can substantially enhance locally deployable model performance while maintaining data privacy, this research provides practical evidence to guide healthcare institutions in selecting and implementing AI systems for clinical decision support.

## Results

### Overall Model Performance and Rankings

**Fig. 1:**
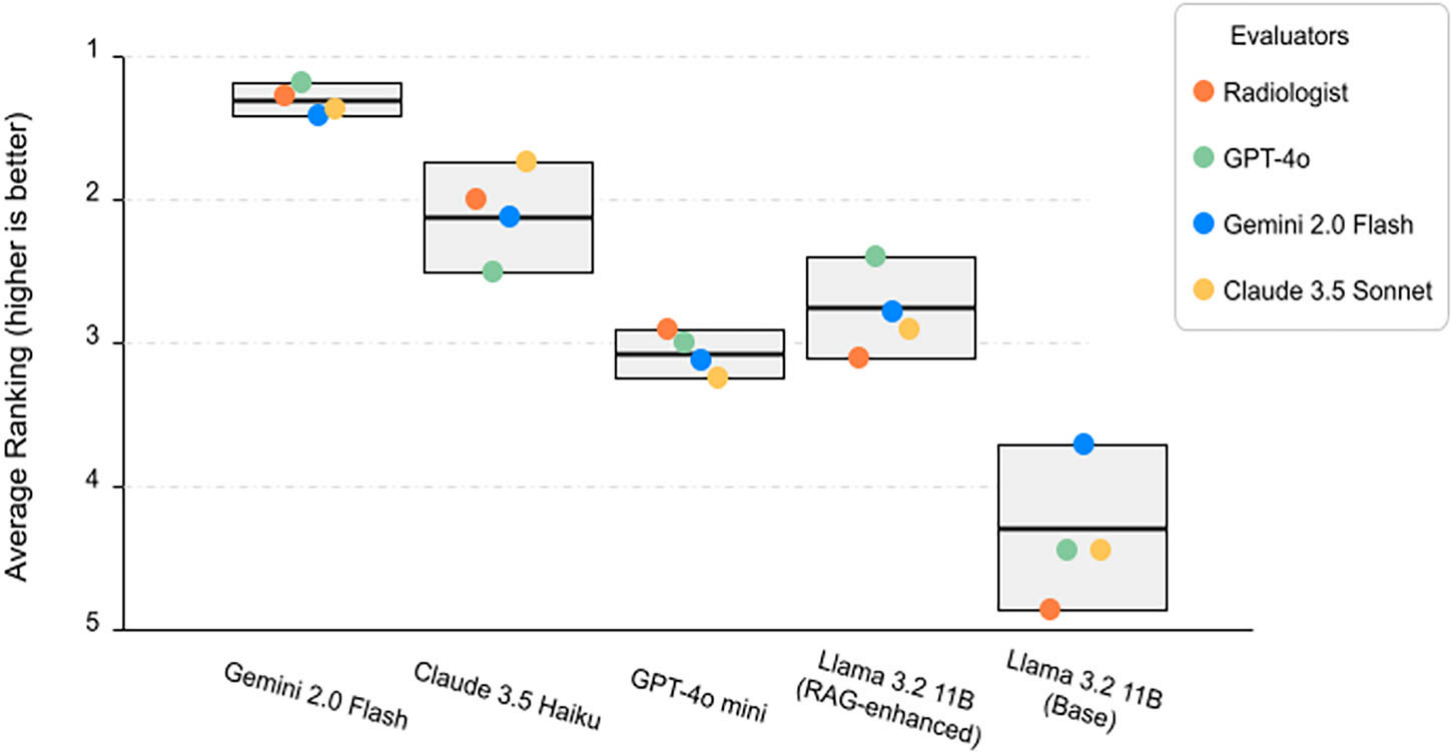
Model Rankings by Human and Automated Evaluators. The mean ranks of five LLMs across 100 ICM consultation scenarios evaluated by a radiologist and three LLM-based scorers, where lower values indicate better performance and error bars represent 95% confidence intervals.

A global Friedman test across the five LLMs confirmed significant differences in median ranks for the 100 iodinated-contrast-media scenarios (χ² = 78.4, p < 0.001). Post-hoc Nemenyi comparisons showed that the three cloud models clustered at the top: Gemini 2.0 Flash was dominant (radiologist mean rank = 1.36; first in 74% of cases), followed by Claude 3.5 Haiku (2.09) and GPT-4o mini (3.21) (Table 1, Fig. 1). The baseline Llama 3.2 11B was ranked last in 86% of cases (mean 4.80). RAG lifted its performance to a mean rank of 3.54, close to GPT-4o mini’s 3.21 (Δ = –1.26; Z = –7.26, p < 0.001). Automated LLM evaluators confirmed the overall cloud supremacy, again ranking Gemini 2.0 Flash and Claude 3.5 Haiku first and second, respectively. Within the remaining models, however, they placed the RAG-enhanced Llama 3.2 11B as 3rd, ahead of GPT-4o mini (Δ = –0.44 to –0.82; Z = +2.56 to +5.43; p ≤ 0.009). This reversal relative to the radiologist’s preference illustrates a systematic divergence between human and LLM-based judgements, primarily due to differential treatment of response time in evaluation frameworks.

**Table 1:** Model Performance Rankings by Radiologist and LLM Evaluators

Evaluator	Model	Mean Rank ± SD	95% CI	1st Place Rate (%)
Radiologist	Gemini 2.0 Flash	1.36 ± 0.72	[1.22, 1.50]	74.0
	Claude 3.5 Haiku	2.09 ± 0.87	[1.92, 2.26]	23.0
	GPT-4o mini	3.21 ± 0.77	[3.06, 3.36]	0.0
	Llama 3.2 11B + RAG	3.54 ± 0.85	[3.37, 3.71]	2.0
	Llama 3.2 11B	4.80 ± 0.60	[4.68, 4.92]	1.0
LLM Evaluator 1	Gemini 2.0 Flash	1.23 ± 0.63	[1.10, 1.36]	87.0
(GPT-4o)	Llama 3.2 11B + RAG	2.53 ± 0.90	[2.35, 2.71]	9.0
	Claude 3.5 Haiku	2.95 ± 1.19	[2.71, 3.19]	3.0
	GPT-4o mini	3.35 ± 1.01	[3.15, 3.55]	4.0
	Llama 3.2 11B	4.32 ± 1.03	[4.11, 4.53]	3.0
LLM Evaluator 2	Gemini 2.0 Flash	1.49 ± 0.80	[1.33, 1.65]	66.0
(Gemini 2.0 Flash Thinking)	Claude 3.5 Haiku	2.25 ± 1.29	[1.99, 2.51]	34.0
	Llama 3.2 11B + RAG	3.12 ± 1.14	[2.89, 3.35]	6.0
	GPT-4o mini	3.56 ± 1.15	[3.33, 3.79]	3.0
	Llama 3.2 11B	3.78 ± 1.28	[3.53, 4.03]	9.0
LLM Evaluator 3	Gemini 2.0 Flash	1.43 ± 0.71	[1.29, 1.57]	65.0
(Claude 3.5 Sonnet)	Claude 3.5 Haiku	1.95 ± 0.98	[1.76, 2.14]	34.0
	Llama 3.2 11B + RAG	3.28 ± 0.95	[3.09, 3.47]	3.0
	GPT-4o mini	3.72 ± 0.88	[3.55, 3.89]	1.0
	Llama 3.2 11B	4.32 ± 0.86	[4.15, 4.49]	1.0

Mean ranks with standard deviations and 95% confidence intervals across 100 ICM consultation scenarios as rated by one radiologist and three LLM-based evaluators. Lower ranks indicate better performance. 1st Place Rate shows percentage of scenarios where each model ranked first. Models are ordered by performance within each evaluator group.

### Inter-Evaluator Agreement and Assessment Patterns

In three LLM evaluators, Claude 3.5 Sonnet exhibited the highest agreement with the radiologist at 82.2%, suggesting its assessments most closely align with human expert judgment. GPT-4o’s evaluation also showed a relatively high agreement rate of 75.5% compared to the radiologist. Across all evaluators, there was broad consensus on the top performer (Gemini 2.0 Flash, 81.6% agreement) and bottom performer (base Llama 3.2 11B), while mid-tier models showed more variable assessments.

The most significant evaluation divergence appeared in mid-tier rankings (Table 1, Fig. 1). All three LLM evaluators consistently ranked the RAG-enhanced Llama 3.2 11B higher than GPT-4o mini, contrary to the radiologist’s assessment (Table 1). This divergence was most pronounced with GPT-4o, which ranked the RAG-enhanced Llama second (mean rank: 2.53) and GPT-4o mini fourth (mean rank: 3.35).

### Clinical Quality and Hallucination Mitigation

Qualitative assessment by a radiologist revealed that hallucinations—particularly in contrast dosing and contraindication recommendations—were present in 8% of responses from the base Llama model and were eliminated (0%) in the RAG-enhanced version (χ²₍Yates₎ = 6.38, p = 0.012; Fisher p = 0.0068). These hallucinations typically involved incorrect ICM protocols, including improper contraindication identification and dosage recommendations, which could potentially impact patient safety (Table 2). None of the cloud-based models exhibited hallucinations in the evaluated scenarios.

**Table 2:** Clinical Errors and Corresponding RAG Corrections in Contrast Media Use

Category	Clinical Scenario	Initial Output Error	RAG Correction
Dosage	Adult contrast dosage	1.5–3.0 mL/kg, max 300 mg iodine/kg	Adjusted to 1.5–2.0 mL/kg, 300 mgI/mL
Dosage	3-year-old child (15 kg)	5–10 mL total dosage	22.5–30 mL (weight-based)
Contraindications	Iodinated contrast risk	Incorrectly mentioned NSF risk	Identified contrast-induced nephropathy
Contraindications	Hyperthyroidism	Omitted thyroid crisis risk	Added crisis risk explanation
Contraindications	Pregnancy and CT	Incomplete information	Added fetal thyroid impact
Contraindications	β-blockers and anaphylaxis	No management advice	Added glucagon use info
Incomplete Orders	Metformin use unknown	Mentioned lactic acidosis risk only	Added stepwise protocol
Avoidable Use	Gallstone suspicion	Recommended contrast CT	Recommended ultrasound first
Avoidable Use	Emphysema, hemorrhage, fracture	Correctly advised no contrast	Maintained accuracy

Summary of initial output errors made by the base LLM, corresponding corrections by the RAG-enhanced model, and whether the errors were fully addressed. Cases are categorized by clinical task type (e.g., dosage, contraindications, appropriateness).

Among the 100 cases, 54% showed marked improvement with RAG—most notably in safety-critical content such as contraindication management and precise dosing guidance—while an additional 33% demonstrated minor gains involving non-critical factual details. These improvements were concentrated in cases that required specialized knowledge of institutional protocols or recently updated guidelines.

### Multidimensional Performance Assessment

**Fig. 2:**
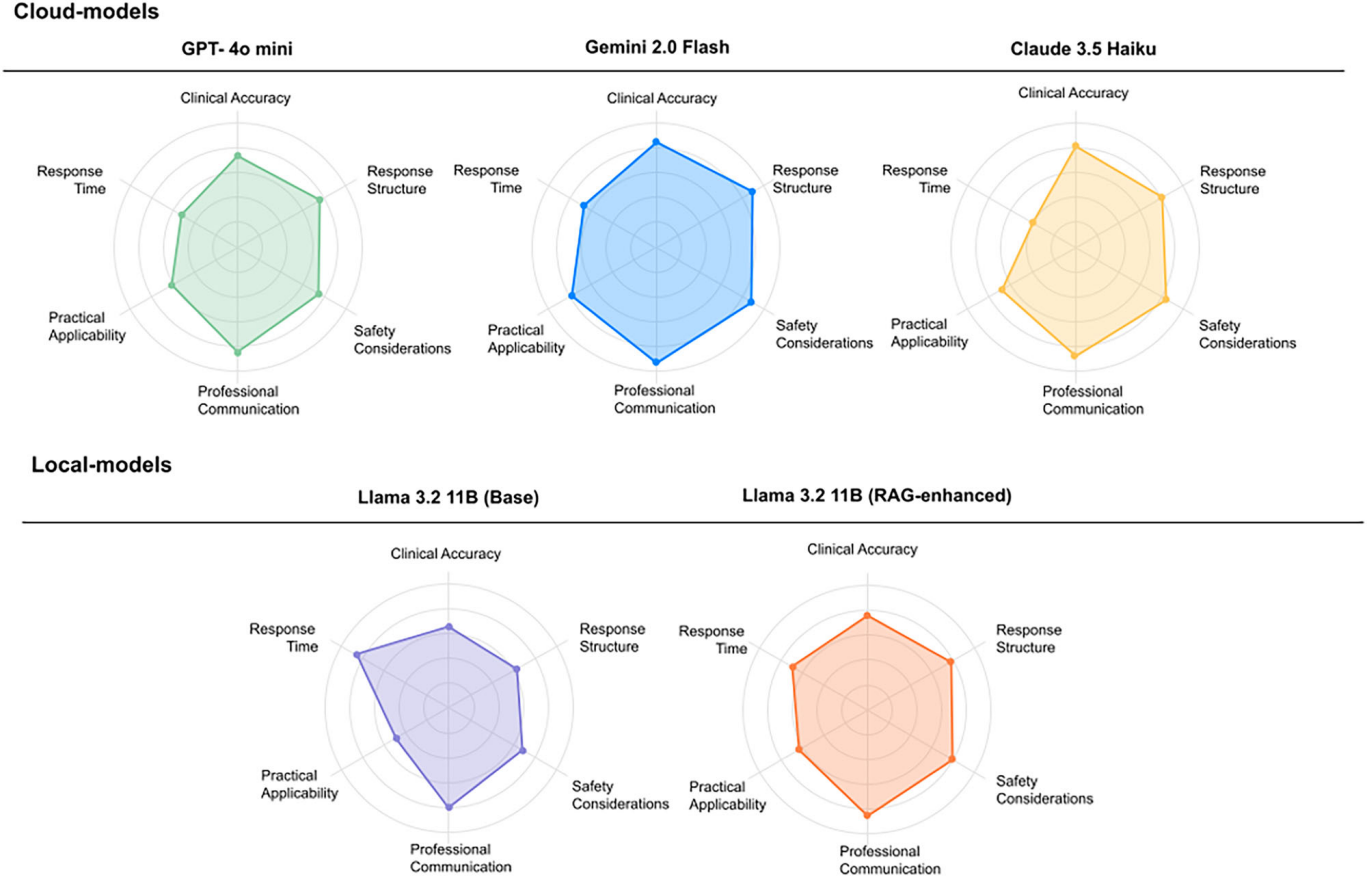
Multidimensional Performance Metrics Across LLMs. Radar charts comparing five LLMs across six key evaluation domains: clinical accuracy, safety, response structure, professional communication, practical applicability, and response time. All performance metrics are normalized to a 0-1 scale for comparative visualization, with 1.0 representing maximum performance in each domain.

Radar chart analysis of LLM evaluations indicated substantial gains in clinical accuracy, safety, and applicability for the RAG-enhanced model, with modest changes in communication and structure (Fig. 2). The most significant improvements occurred in domains directly related to patient safety and clinical decision-making.

Specifically, clinical accuracy scores improved from a mean of 14.2 to 21.5 points (out of 25), and safety considerations improved from 11.8 to 18.7 points (out of 20). Professional communication showed moderate enhancement (13.4 to 16.8 out of 20), while response structure remained relatively stable (7.8 to 8.3 out of 10), as the base model already demonstrated adequate formatting capabilities.

### Response Time Analysis

**Fig. 3:**
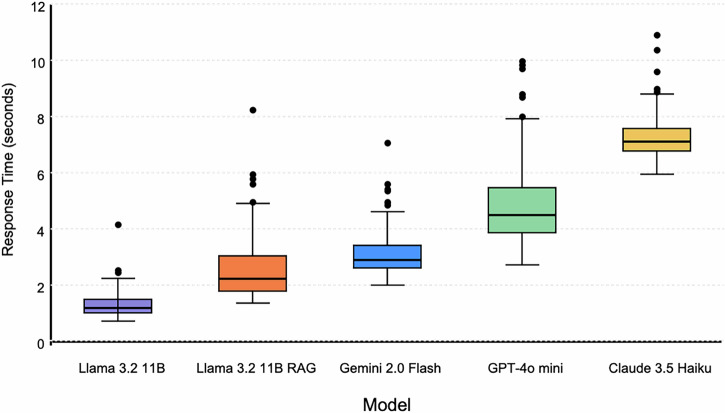
Comparative Response Times of Evaluated LLMs. Boxplots showing response time distributions for five LLMs where each point represents an individual measurement, center lines show medians, box edges show 25th and 75th percentiles, and whiskers extend to the most extreme data points within 1.5 × IQR from the box edges.

In terms of response time, the RAG-enhanced model (2.58 s) remained faster on average than all cloud-based models (GPT-4o mini: 4.87 s, Claude 3.5 Haiku: 7.25 s, Gemini 2.0 Flash: 3.14 s), despite increased latency compared to its base version (1.31 s) (Fig. 3). The difference was statistically significant for all comparisons, including Gemini 2.0 Flash (t = –3.96, p = 0.00011).

Response time variability differed notably across models. Claude 3.5 Haiku demonstrated the most consistent performance with a coefficient of variation (CV) of 10.76%, while the RAG-enhanced Llama 3.2 11B showed the highest variability (CV = 44.19%), likely due to fluctuations in knowledge retrieval latency. The base Llama 3.2 11B (CV = 37.40%), GPT-4o mini (CV = 31.21%), and Gemini 2.0 Flash (CV = 26.43%) exhibited intermediate variability. While the RAG-enhanced model’s response time was more variable, the trade-off was acceptable given the substantial performance improvements in clinical accuracy and safety. This response time advantage contributed to higher automated evaluation rankings compared to human expert preferences. Task complexity showed minimal impact on response times across models, with variability primarily driven by architecture-specific characteristics rather than scenario complexity.

## Discussion

This study demonstrates that a retrieval-augmented local LLM can achieve clinically reliable performance in a safety-critical radiological use case while preserving patient privacy. By integrating curated guideline-based knowledge into a lightweight LLM, we observed significant improvements in clinical accuracy, risk stratification, and communication quality—criteria that are central to iodinated contrast media (ICM) decision-making. Notably, the RAG-enhanced model eliminated hallucinations observed in the base model and achieved faster average response times than all evaluated cloud-based LLMs, suggesting feasibility for real-time use in clinical settings.

Our findings align with recent research demonstrating RAG’s potential to enhance LLM performance in specialized medical domains. Gupta et al. (2024) emphasized RAG’s application in medical knowledge retrieval, showing improvements in accuracy and reliability^15^. Mortaheb et al. (2025) introduced a multimodal evaluation framework ensuring contextual relevance^9^, while Nguyen et al. (2024) reported significant accuracy improvements in clinical question-answering when incorporating RAG into language models^8^. These studies collectively confirm that integrating domain-specific knowledge improves evidence-based reasoning capabilities of LLMs, leading to enhanced accuracy, factual consistency, and clinical relevance in radiological applications.

Our findings reinforce RAG’s critical role in hallucination mitigation, a fundamental requirement for deploying AI systems in clinical workflows. The complete elimination of hallucinations (reducing incidence from 8% to 0%) in our RAG-enhanced model represents a notable achievement, especially considering previous studies reporting hallucination rates of 28.6-39.6% in medical-specialized models^16^. Incorrect recommendations, particularly in contraindication management and contrast dosing, pose substantial patient safety risks. The success of our approach likely stems from developing a robust knowledge base centered specifically on ICM consultation, enabling the model to generate responses with clear evidential support. This achievement is particularly significant for safety-critical applications where even occasional hallucinations could potentially endanger patients. However, it is crucial to emphasize that “zero hallucinations” indicates absence of clinically dangerous misinformation rather than perfect clinical responses. Final clinical judgment, risk-benefit assessment, and treatment decisions must always remain with qualified radiologists who integrate AI-generated information with patient-specific factors, clinical experience, and comprehensive medical context. RAG enhancement improves the reliability of supportive information but does not replace human clinical expertise in patient care decisions.

Given the legal and ethical constraints associated with cloud-based LLMs, particularly regarding PHI transmission under HIPAA and GDPR frameworks, our results suggest that RAG-enhanced locally deployable models provide a viable alternative that balances safety, speed, and privacy^4,17^. By processing data entirely within institutional networks, locally deployed LLMs substantially reduce information leakage risks, addressing a primary concern in healthcare AI adoption. Our on-premise deployment validation (Supplementary Note 1) demonstrates competitive performance while maintaining complete data privacy within institutional infrastructure. While locally deployed models have traditionally faced performance limitations due to size constraints, our RAG-enhanced Llama 3.2 11B demonstrates that clinically useful AI support can be achieved in privacy-sensitive healthcare environments without compromising on essential performance metrics. This addresses the fundamental privacy-performance dilemma highlighted in our introduction, providing practical evidence for healthcare institutions evaluating AI implementation strategies.

The observed divergence between human and LLM-based evaluators highlights an important consideration for future benchmarking frameworks. While LLM-based evaluators consistently ranked the RAG-enhanced Llama 3.2 11B higher than GPT-4o mini, the radiologist favored the latter. This divergence primarily stems from differential treatment of response time in evaluation frameworks. Human radiologists excluded response time from ranking criteria, focusing solely on clinical quality, while LLM evaluators included response time as a weighted component. The RAG-enhanced model’s superior speed (2.58 s vs 4.87 s for GPT-4o mini) contributed to higher automated rankings despite comparable clinical quality scores, reflecting the difference between clinical priority (accuracy over speed) and system performance evaluation (efficiency inclusion). Our analysis reported mean ranks, standard deviations, and 95% confidence intervals to complement the ordinal ranking data. Although such metrics derive from non-interval data, they are widely used in recent LLM benchmarking studies (e.g., MT-Bench, Chatbot Arena) as descriptive evaluator preference and ranking consistency indicators. These metrics provide a practical and interpretable summary of comparative model performance when interpreted with non-parametric tests and complete rank distributions.

This divergence between evaluation methodologies underscores the need for hybrid evaluation strategies that combine quantitative and qualitative metrics, especially for high-stakes applications. Recent research has highlighted both the potential and limitations of automated LLM evaluation approaches in medical contexts^18,19^. Research on emergency medicine summary generation has shown that LLM-generated content rated highly by automated evaluations may contain subtle clinical utility and safety deficiencies only identifiable through expert clinician review^20^. Our hybrid approach offers valuable insights for refining methodologies used to evaluate LLMs in clinical contexts.

Several important limitations merit consideration. First, our controlled synthetic scenarios may not capture the full complexity of real-world clinical consultations, including incomplete patient information and diagnostic uncertainty, and time-sensitive decision-making under clinical pressure. The observed zero hallucination rate in cloud-based models likely reflects this controlled environment rather than performance under challenging clinical conditions. Second, evaluation by a single radiologist may limit the generalizability of our human expert rankings and hallucination detection, though our multi-evaluator validation (Supplementary Note 2) demonstrates high inter-rater agreement across different experience levels. Third, our temperature parameter selection (0.2) represents a practical compromise due to documented Llama model instability at temperature 0.0, though systematic sensitivity analysis (Supplementary Note 3) validates that clinical quality metrics remain statistically unchanged while reproducibility shows manageable reduction.

These limitations can be effectively addressed through appropriate clinical implementation strategies: such as radiologists serving as essential validation intermediaries for AI-generated recommendations (human in the loop), advancing RAG and self-refinement technologies for enhanced robustness, and multi-layer quality assurance protocols integrated within existing clinical workflows. While our synthetic evaluation provides essential baseline performance data and methodological frameworks, these implementation strategies suggest that effective clinical deployment remains viable with appropriate human oversight and technological enhancements. Future research should prioritize real-world validation using enhanced implementation frameworks, systematic RAG knowledge base maintenance and updating protocols, expansion to multiple expert reviewers across different subspecialties, and comprehensive testing in active clinical environments with appropriate privacy safeguards.

This study demonstrates that RAG-enhanced local LLMs can provide meaningful clinical decision support under controlled conditions. While our approach reveals inherent limitations, it offers a viable pathway for privacy-preserving clinical AI deployment with appropriate human oversight. As AI technologies advance, the principles demonstrated here will support more robust clinical applications while maintaining privacy advantages of local deployment.

## Methods

### Consultation Scenarios Development

We designed 100 simulated consultation scenarios covering a diverse range of iodinated contrast media (ICM) use cases, including appropriateness, contraindications, incomplete orders, and avoidable contrast use. These scenarios systematically reflected five key categories in radiological practice: (1) appropriateness consultations for specific clinical indications, (2) optimal contrast agent selection and protocols, (3) contraindication identification and risk assessment, (4) recognition of incomplete ordering information, and (5) identification of cases where contrast could be avoided.

Scenarios were initially generated using GPT-4o and Claude 3.5 Sonnet with structured prompts for clinical authenticity. To ensure realism and guideline compliance, two radiologists (4 and 30 years of experience, respectively) independently reviewed and revised each scenario according to current ACR and ESUR guidelines. Special attention was given to incorporating realistic clinical nuances and decision-making challenges commonly encountered in ICM consultations. The complete set of scenarios is available in Supplementary Data 1.

### Language Models Configuration

We benchmarked five LLMs—three cloud-based models (GPT-4o mini from OpenAI, Gemini 2.0 Flash from Google, and Claude 3.5 Haiku from Anthropic) accessed through their public REST APIs, and two locally deployable models (Llama 3.2 11B from Meta, as a baseline and RAG-enhanced variant). Llama 3.2 11B is designed as a locally deployable, lightweight parameter model ideal for on-premises computing environments.

**Fig. 4:**
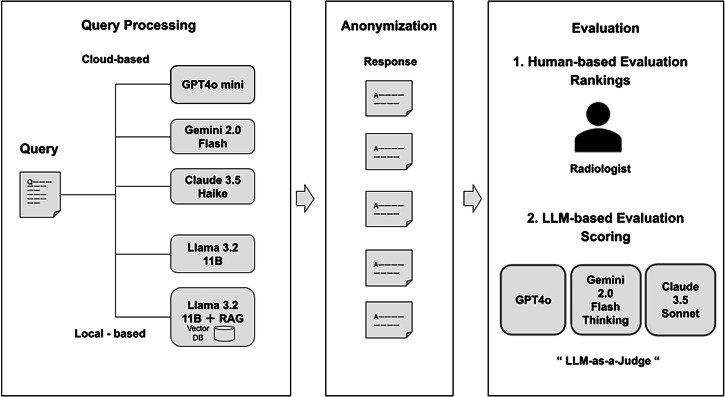
Evaluation Workflow for Language Model Responses. Schematic of the evaluation pipeline. Each clinical query was simultaneously processed by five LLMs (three cloud-based and two locally deployable models). The generated responses were anonymized and evaluated by a radiologist and three LLM-based evaluators using both human ranking and rubric-based scoring.

This study deployed the Llama 3.2 11B via GroqCloud’s hosted inference API to facilitate direct performance comparisons with the cloud-native models under controlled conditions (Fig. 4). To address concerns regarding true privacy-preserving deployment, we conducted additional validation using dedicated enterprise hardware (HP Z8 Fury G5 with NVIDIA RTX 6000 Ada Generation, 48GB VRAM; Intel Xeon W7-3445, 20 cores; 512GB DDR5 RAM), with results detailed in Supplementary Note 1.

All models were deployed via the Dify platform (version 0.10.0) with standardized parameters, including consistent temperature settings (0.2) and single-shot response generation^21^. Temperature parameter selection represents a practical compromise due to documented Llama model instability at temperature 0.0, with comprehensive sensitivity analysis detailed in Supplementary Note 3. For each model, we recorded both the responses and the response times while maintaining all other default settings for consistent comparison.

For the automated evaluation process, we employed three high-performance models (GPT-4o, Gemini 2.0 Flash Thinking, and Claude 3.5 Sonnet) as evaluators. Table 3 documents the technical specifications for all models used in this study, including parameter sizes, deployment characteristics, and implementation details.

**Table 3:** Technical Specifications of Evaluated Language Models for ICM Consultation and Response Evaluation

LLM Name	Use Case	Deployment	Developer	Model Scale	Description	Availability
GPT-4o mini	ICM Consultation	Cloud	OpenAI	Large (MoE)	Optimized for speed	2024-07
Gemini 2.0 Flash	ICM Consultation	Cloud	Google	Large	Fast-response model	2024-12
Claude 3.5 Haiku	ICM Consultation	Cloud	Anthropic	Medium–Large	Lightweight model	2024-11
Llama 3.2 11B	ICM Consultation	Local	Meta	11B	Base on-premise model	2024-09
GPT-4o	Evaluator	Cloud	OpenAI	Very Large	Full-scale evaluator	2024-05
Gemini 2.0 Flash Thinking	Evaluator	Cloud	Google	Very Large	Reasoning-tuned version	2024-12
Claude 3.5 Sonnet	Evaluator	Cloud	Anthropic	Very Large	Mid-tier evaluator	2024-06

**Note:** Deployment — Cloud = vendor-hosted API only; Local = weights are deployable on-premise, though in this study Meta Llama 3.2 11B was run on GroqCloud (TLS-encrypted, no-retention) through the same Dify pipeline for parity. Model scale — “Large” ≈ tens-to-hundreds B parameters, “Very Large” the upper end; Llama’s 11 B count is official. “MoE” = Mixture-of-Experts architecture.

### Retrieval-Augmented Generation Implementation

For the RAG-enhanced model, we compiled a knowledge base from authoritative sources, including the ACR Manual on Contrast Media, ESUR guidelines, institutional protocols, and relevant literature^22–24^. Knowledge entries were initially generated in a question-answer format using Claude 3.5 Sonnet and were subsequently reviewed, modified, and organized by two radiologists (Table 4).

**Table 4:** Sample Knowledge Entry from the RAG Knowledge Base

Topic	Question-Answer Format
Precautions for Contrast Media Administration in Patients with Renal Dysfunction	Q: What are the important precautions and considerations when administering contrast media to patients with renal dysfunction? A: • Pre-existing renal dysfunction is considered a risk factor for acute renal failure caused by iodinated contrast media, with eGFR now commonly used as the preferred indicator of renal function - Patients with eGFR below 30 mL/min/1.73 m² face significantly increased risk and require sufficient preventive measures - Contrast-induced nephropathy (CIN) is defined as an increase in serum creatinine level by 0.5 mg/dL or 25% or more from baseline within 72 hours after contrast media administration - Key risk factors for CIN include advanced age, diabetes with chronic kidney disease (CKD), use of diuretics (especially loop diuretics), and use of NSAIDs - Appropriate hydration protocols using normal saline solution or sodium bicarbonate solution are recommended as preventive measures - Repeated contrast-enhanced CT scans within a short period (24-48 hours) should be avoided

Example of a structured entry used in the retrieval-augmented generation (RAG) system, formatted as a clinical question and answer. This entry addresses precautions for administering contrast media to patients with renal dysfunction.

The knowledge base was structured as segments with a maximum of 600 tokens, comprising 66 chunks with an average of 337 characters per segment. These segments were transformed into high-dimensional embeddings using OpenAI’s text-embedding-3-large model and indexed in “High Quality” mode. The system employed a hybrid search method that combined semantic vector search with keyword-based retrieval.

**Fig. 5:**
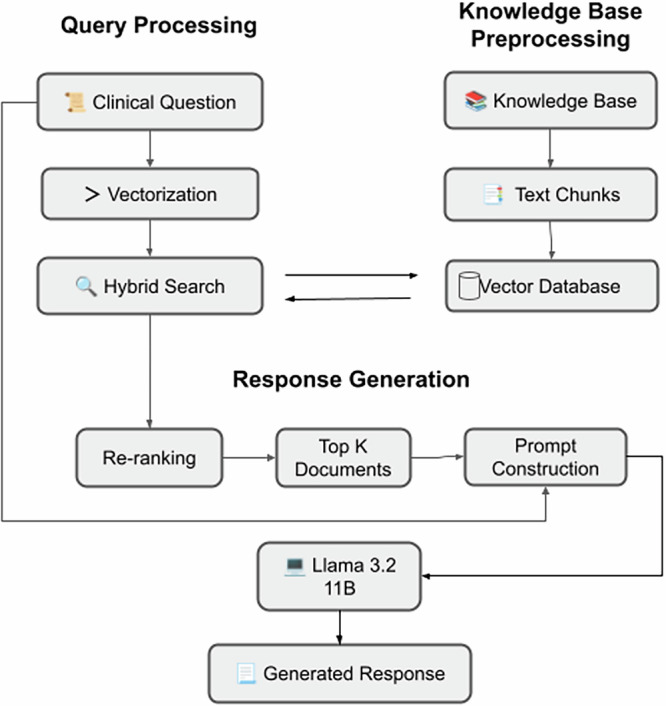
Retrieval-Augmented Generation (RAG) Pipeline for Local LLMs. Diagram illustrating the RAG implementation. Domain-specific knowledge is embedded and indexed using semantic and keyword-based retrieval. Retrieved context chunks are ranked and integrated into the prompt before inference by the local LLM.

For each clinical query, the system retrieved four relevant context fragments (TopK =4 ) based on cosine similarity calculations. These retrieved fragments were then ranked according to their relevance scores and incorporated into the prompt structure for the Llama 3.2 11B model (Fig. 5).

### Evaluation Methodology

We used a two-tier evaluation strategy: (i) human expert review by a board-certified radiologist, and (ii) automated scoring by three large LLM “judges.”

For the human tier, the radiologist reviewed all responses in a blinded manner, ranking the anonymized responses from 1st (best) to 5th (worst) based on clinical appropriateness and practical applicability, without considering response time. In addition to rubric-based scoring, each response was screened for hallucinations, defined as clinically incorrect or guideline-inconsistent statements that could alter patient management (e.g., wrong dosage, missing contraindication). Hallucination presence (yes/no) was recorded per scenario, and the frequencies were compared between models using χ² (with Yates correction) and Fisher’s exact tests.

For automated LLM “judge” evaluation, we utilized three high-performance LLM evaluators within a G-EVAL-like framework^25^. Each response was systematically compared against reference standards developed by radiology experts, using six predefined criteria:

1. Clinical Accuracy: Alignment with established medical guidelines and factual correctness.
2. Safety: Proper identification of contraindications and emphasis on patient safety measures.
3. Response Structure: Clarity and logical organization of the answer.
4. Professional Communication: Appropriate use of medical terminology and professional language.
5. Practical Applicability: Provision of actionable clinical recommendations.
6. Response Time: Performance against predefined speed thresholds.

LLM evaluators assessed the responses based on these criteria, with final rankings determined by the total combined score. In cases of tied scores, models were assigned the same higher rank. Comprehensive details regarding the evaluation prompts and scoring rubrics are available in Supplementary Note 4.

### Statistical Analysis

All analyses were conducted in Python 3.9.13 with scipy 1.10.0 and statsmodels 0.13.5. Because the Shapiro–Wilk test revealed significant departures from normality (p < 0.05) in the rank distributions, we exclusively applied non-parametric procedures. Overall differences among the five LLMs were examined with the Friedman test, which accounts for the repeated-measures nature of the 100 shared consultation scenarios. Pairwise contrasts were then assessed with the Nemenyi post-hoc test; for the two comparisons of primary interest—GPT-4o mini versus the RAG-enhanced Llama, and baseline versus RAG Llama—we also report Mann-Whitney U (Wilcoxon rank-sum) statistics with Bonferroni-adjusted significance thresholds. Effect sizes are expressed as the difference in mean ranks (Δ), accompanied by Wilcoxon Z values and two-sided p values. Descriptively, we present mean rank ± standard deviation and 95% confidence intervals to visualise performance spread and inter-evaluator agreement, following conventions established in recent LLM benchmarks such as MT-Bench and Chatbot Arena.

### Ethical Considerations

This study did not require Institutional Review Board approval as it utilized simulated clinical scenarios rather than actual patient data. All consultations were fictional cases designed to reflect common clinical situations without incorporating any protected health information.

## Supplementary information

Supplementary information

Supplementary Data 1

Supplementary Data 2

## Data Availability

The complete dataset of 100 synthetic clinical scenarios used in this study is provided as Supplementary Data 1. The dataset includes scenario IDs, clinical inquiries, primary categories, and scenario types across four main clinical domains: proper contrast agent usage and protocols (11 scenarios), contraindication and risk management (32 scenarios), appropriateness consultation across multiple specialties (37 scenarios), and order deficiency management (20 scenarios). Model response data supporting the conclusions are available from the corresponding author upon reasonable request to protect potential intellectual property considerations.

## Code availability

Custom evaluation scripts for LLM response assessment and RAG implementation code are available from the corresponding author upon reasonable request. The study utilized standard open-source software packages: Python 3.9.13, scipy 1.10.0, statsmodels 0.13.5, and the Dify platform (version 0.10.0) for model deployment. No proprietary code was developed that would require special access restrictions.

## References

[1] Liu, Z. et al. Radiology-GPT: A Large Language Model for Radiology. Preprint at arXiv:2306.08666 (2023).

[2] Nakaura, T. et al. The impact of large language models on radiology: a guide for radiologists on the latest innovations in AI. *Jpn. J. Radiol.* **37**, 1–12 (2024). 10.1007/s11604-024-01552-0 PMC11217134 38551772

[3] Wada, A. et al. Optimizing GPT-4 Turbo Diagnostic Accuracy in Neuroradiology through Prompt Engineering and Confidence Thresholds. *Diagnostics* **14**, 1541 (2024). 39061677 10.3390/diagnostics14141541 PMC11276551

[4] Abbasi, N. & Smith, D. A. Cybersecurity in Healthcare: Securing Patient Health Information (PHI). *J. Knowl. Learn. Sci. Technol.* **3**, 278–287 (2024).

[5] Mohan, A. et al. Securing AI Inference in the Cloud: Is CPU-GPU Confidential Computing Ready? In Proc. 17th IEEE Int. Conf. Cloud Comput. 164-175 (IEEE, 2024).

[6] Bedi, S. et al. A Systematic Review of Testing and Evaluation of Healthcare Applications of Large Language Models (LLMs). Preprint at medRxiv:2024.04.15.24305869 (2024).

[7] Lewis, P. et al. Retrieval-Augmented Generation for Knowledge-Intensive NLP Tasks. *Advances in Neural Information Processing Systems* **33**, 9459–9474 (2020).

[8] Nguyen, Q. et al. Advancing Question-Answering in Ophthalmology with Retrieval-Augmented Generation (RAG): Benchmarking Open-source and Proprietary Large Language Models. Preprint at medRxiv:2024.11.18.24317510 (2024). 10.1167/tvst.14.9.18 PMC12439504 40938068

[9] Mortaheb, M., Khojastepour, M. A. A., Chakradhar, S. T. & Ulukus, S. RAG-Check: Evaluating Multimodal Retrieval Augmented Generation Performance. Preprint at arXiv:2501.03995 (2025).

[10] Telenti, A. et al. Large language models for science and medicine. *Eur. J. Clin. Investig.* **54**, e14183 (2024). 38381530 10.1111/eci.14183

[11] Davenport, M. S., Chu, P., Szczykutowicz, T. P. & Smith-Bindman, R. Comparison of Strategies to Conserve Iodinated Intravascular Contrast Media for Computed Tomography During a Shortage. *JAMA* **328**, 476–478 (2022). 35679081 10.1001/jama.2022.9879 PMC9185519

[12] CADTH. Optimizing the Use of Iodinated Contrast Media for CT: Managing Shortages and Planning for a Sustainable and Secure Supply. CADTH report 613 (2023). 37883604

[13] Sessa, M. et al. Campania preventability assessment committee: a focus on the preventability of the contrast media adverse drug reactions. *Expert Opin. Drug Saf.* **15**, 51–59 (2016). 27855534 10.1080/14740338.2016.1226280

[14] Siewert, B., Brook, O. R., Hochman, M. & Eisenberg, R. L. Impact of Communication Errors in Radiology on Patient Care, Customer Satisfaction, and Work-Flow Efficiency. *Am. J. Roentgenol.* **206**, 573–579 (2016). 26901014 10.2214/AJR.15.15117

[15] Gupta, S., Ranjan, R. & Singh, S. N. A Comprehensive Survey of Retrieval-Augmented Generation. Preprint at arXiv:2410.12837 (2024).

[16] Chelli, M. et al. Hallucination Rates and Reference Accuracy of ChatGPT and Bard for Systematic Reviews: Comparative Analysis. *J. Med. Internet Res.* **26**, e53164 (2024). 38776130 10.2196/53164 PMC11153973

[17] Ng, M. Y., Helzer, J., Pfeffer, M. A., Seto, T. & Hernandez-Boussard, T. Development of secure infrastructure for advancing generative artificial intelligence research in healthcare at an academic medical center. *J. Am. Med. Inform. Assoc.* **32**, 586–588 (2025). 39836496 10.1093/jamia/ocaf005 PMC11833461

[18] Iglesia, I. D. et al. Ranking Over Scoring: Towards Reliable and Robust Automated Evaluation of LLM-Generated Medical Explanatory Arguments. In *Proc. 31st Int. Conf. Computational Linguistics (COLING)*, 2025.coling-main.634 (2025).

[19] Gu, J. et al. A Survey on LLM-as-a-Judge. Preprint at arXiv:2411.15594 (2024).

[20] Szymanski, A. et al. Limitations of the LLM-as-a-Judge Approach for Evaluating LLM Outputs in Expert Knowledge Tasks. In Proc. 30th Int. Conf. Intelligent User Interfaces (IUI), 952−966. (ACM, 2025). 10.1145/3708359.3712091.

[21] Dify.AI. The Innovation Engine for Generative AI Applications. https://dify.ai/ (2023).

[22] Beckett, K. R., Moriarity, A. K. & Langer, J. M. Safe Use of Contrast Media: What the Radiologist Needs to Know. *Radiographics* **35**, 1738–1750 (2015). 26466182 10.1148/rg.2015150033

[23] Isaka, Y. et al. Guideline on the use of iodinated contrast media in patients with kidney disease 2018. *Clin. Exp. Nephrol.* **24**, 1–44 (2020). 31709463 10.1007/s10157-019-01750-5 PMC6949208

[24] Thomsen, H. S. & Morcos, S. K. & ESUR. ESUR guidelines on contrast media. *Abdom. Imaging* **31**, 131–140 (2006). 16447092 10.1007/s00261-005-0380-y

[25] Liu, Y. et al. G-Eval: NLG Evaluation using GPT-4 with Better Human Alignment. *In Proc. Conf. Empirical Methods in Natural Language Processing (EMNLP)*, 2511–2522 (2023).

