# Supplementary material for: Retrieval-augmented generation elevates local LLM quality in radiology contrast media consultation
Source: NPJ Digit Med. 2025 Jul 2;8:395. doi: 10.1038/s41746-025-01802-z (PMC12223273; doi:10.1038/s41746-025-01802-z)
Supplement: Supplementary file 1 — Supplementary information [file 41746_2025_1802_MOESM1_ESM.pdf]

Supplementary Information

Retrieval-Augmented Generation Elevates Local LLM Quality in Radiology Contrast-Media Consultation

---

Supplementary Note 1: On-Premise Deployment Performance Validation

Background

To address concerns regarding true on-premise deployment validation, we conducted additional experiments using dedicated local hardware infrastructure to validate privacy-preserving clinical AI performance.

Local Hardware Configuration

Workstation Specifications:

- **Model**: HP Z8 Fury G5 Enterprise Workstation
- **CPU**: Intel Xeon W7-3445 (2.6-4.8GHz, 20 cores)
- **Memory**: 512GB DDR5 RAM
- **GPU**: NVIDIA RTX 6000 Ada Generation (48GB VRAM)
- **Storage**: 2TB NVMe SSD
- **OS**: Windows 11 Pro for Workstations

Software Environment:

- **Platform**: Ollama (local deployment)
- **Model**: llama3.2-vision:11b
- **RAG Implementation**: Local vector database with hybrid search
- **Embedding**: text-embedding-3-large (local deployment)

Performance Comparison Results

Supplementary Table 1: Cloud vs On-Premise Performance (n=22 trials)

Component	Mean Time (s)	Median (s)	SD (s)	CV (%)	Range (s)
**Cloud Environment**					
Llama 3.2 11B (GroqCloud)	1.31	1.20	0.49	37.4	0.72 – 4.14

Llama 3.2 11B + RAG (GroqCloud)	2.58	2.25	1.14	44.2	1.35 – 8.24
**On-Premise Environment**					
Llama 3.2 11B (Local)	2.15	1.95	0.78	36.3	1.12 – 4.68
Llama 3.2 11B + RAG (Local)	3.34	2.89	1.33	39.8	1.85 – 6.95

SD = Standard Deviation; CV = Coefficient of Variation

Findings and Clinical Implications

Local deployment achieved competitive performance with mean response time of 3.34s, remaining faster than cloud-based competitors (GPT-4o mini: 4.87s, Claude 3.5 Haiku: 7.25s) while maintaining complete patient privacy protection through data containment within institutional infrastructure. The 1.3-fold response time increase compared to cloud deployment (2.58s → 3.34s) represents acceptable trade-off for privacy-preserving clinical AI, with performance remaining within clinically acceptable ranges for routine ICM consultations. This validation confirms the feasibility of truly local, privacy-preserving clinical AI systems suitable for deployment in real-world healthcare settings.

Supplementary Note 2: Multi-Radiologist Performance Evaluation

Methodology

To address concerns regarding single-evaluator limitations, we conducted expanded evaluation with three radiologists with diverse experience levels: two board-certified radiologists (28 years of clinical experience and 20 years of clinical experience), and a senior resident (4 years of clinical experience). Each radiologist independently evaluated 20 representative ICM consultation scenarios in a blinded, randomized fashion using standardized ranking criteria: clinical appropriateness, safety considerations, practical applicability, communication clarity, and guideline adherence.

Overall Performance Rankings

Supplementary Table 2: Model Performance Rankings by Three Independent Radiologist Evaluators (n=66 evaluations)

Model	Mean Rank ± SD	Median	95% CI	1st Place Rate (%)	Last Place Rate (%)

Gemini 2.0 Flash	1.37 ± 0.49	1.0	1.24-1.49	71.7	0
Claude 3.5 Haiku	2.08 ± 0.70	2.0	1.90-2.26	25.0	0
GPT-4o mini	3.20 ± 0.88	3.0	2.97-3.43	3.3	0
Llama 3.2 + RAG	3.75 ± 0.63	4.0	3.59-3.91	0	8.3
Llama 3.2 11B	4.80 ± 0.40	5.0	4.70-4.90	0	91.7

SD = Standard Deviation; CI = Confidence Interval

Inter-Evaluator Reliability

Supplementary Table 3: Inter-Evaluator Agreement Statistics

Measure	Value	Interpretation
Kendall's Coefficient of Concordance (W)	0.78	High agreement
Average Spearman Correlation	0.81	Strong correlation
Perfect Agreement Cases	8/20 (40%)	Moderate concordance
Within 1-Rank Difference	17/20 (85%)	High practical agreement

Pairwise Correlations:

- Senior-Attending Level (A vs B): $r = 0.84$, $p < 0.001$
- Senior-Resident Level (A vs C): $r = 0.79$, $p < 0.001$
- Attending-Resident Level (B vs C): $r = 0.80$, $p < 0.001$

Experience-Based Evaluation Patterns

Individual Evaluator Characteristics:

- **Senior Radiologist (28y)**: Conservative, safety-oriented; most consistent rankings (CV = 22.4%)
- **Mid-Career Radiologist (20y)**: Balanced efficiency-thoroughness; moderate innovation tolerance
- **Resident Radiologist (4y)**: Evidence-based, guideline-adherent; highest variability (CV = 31.2%)

All three evaluators showed consistent model preferences: Gemini 2.0 Flash (70-75% first place), Claude 3.5 Haiku (25% first place), with strong agreement on top and bottom performers while showing moderate variation in mid-tier rankings.

Clinical Implications

The high inter-evaluator agreement (W = 0.78) despite diverse experience levels confirms that model performance differences are clinically meaningful across expertise levels and that RAG enhancement effects are recognizable to practicing radiologists regardless of experience. The consistent ranking patterns provide confidence for clinical deployment decisions based on expert evaluation and validate the reliability of single-expert evaluations in similar clinical AI assessment contexts.

This multi-evaluator validation strengthens confidence in our primary conclusions regarding RAG-enhanced local LLM performance while demonstrating that the observed model performance differences represent clinically meaningful distinctions that transcend individual evaluator characteristics.

Supplementary Note 3: Temperature Parameter Sensitivity Analysis

Background

To ensure stable output across architectures, the main study used a conservative temperature setting of 0.2. We conducted a post-hoc analysis to assess how temperature influences clinical quality and reproducibility in GPT-4o-mini and Llama 3.2 11B + RAG.

Methods

- **Scenarios**: 22 new ICM cases; The complete set of scenarios is available in Supplementary Data 2.
- **Models**: GPT-4o-mini, Llama 3.2 11B + RAG
- **Temperatures**: GPT-4o-mini: 0.0, 0.2, 0.5; Llama+RAG: 0.01, 0.2, 0.5
- **Metrics**: Accuracy, Safety, Structure, Tone, Applicability, Reproducibility (1–5 scale)
- **Evaluation**: LLM-as-a-Judge (OpenAI o3)
- **Analysis**: Wilcoxon signed-rank test, Bonferroni correction; Cohen's d

Results

Supplementary Table 4: Clinical Quality Scores by Temperature Setting (Mean ± SD)

Model	Temp	Accuracy	Safety	Structure	Tone	Applicability	Reproducibility
**GPT-4o-mini**	0.0	3.73 ± 0.55	4.18 ± 0.40	4.00 ± 0.00	4.05 ± 0.21	3.45 ± 0.51	4.73 ± 0.65

	0.2	3.73 ± 0.55	4.27 ± 0.46	4.00 ± 0.00	4.09 ± 0.29	3.45 ± 0.51	4.09 ± 0.43
	0.5	3.05 ± 0.58	3.82 ± 0.50	3.91 ± 0.29	3.95 ± 0.38	3.18 ± 0.59	3.36 ± 0.49
**Llama+ RAG**	0.01	3.59 ± 0.67	4.00 ± 0.69	3.95 ± 0.21	4.05 ± 0.21	3.32 ± 0.72	4.55 ± 0.51
	0.2	3.50 ± 0.60	3.95 ± 0.58	3.86 ± 0.35	4.09 ± 0.29	3.41 ± 0.73	3.91 ± 0.53
	0.5	2.95 ± 0.72	3.55 ± 0.67	3.73 ± 0.46	3.91 ± 0.43	3.09 ± 0.81	3.18 ± 0.50

Supplementary Table 5: Significant Differences in Reproducibility (Wilcoxon Test + Cohen's d)

Model	Comparison	Δ (Mean)	p	d	Significant
GPT-4o-mini	0.0 vs 0.2	-0.64	<0.001	–1.11	Yes
	0.2 vs 0.5	-0.73	<0.001	–1.58	Yes
Llama+ RAG	0.01 vs 0.2	-0.64	0.001	–1.24	Yes
	0.2 vs 0.5	-0.73	<0.001	–1.41	Yes

Interpretation and Implications

Clinical quality is preserved at temperature ≤ 0.2 for both models, while reproducibility significantly degrades at ≥ 0.5 across architectures. We recommend temperature 0.0 for GPT-family models, 0.01 for Llama-family models, and avoiding ≥ 0.5. Our findings confirm that the temperature setting (0.2) used in the main study was conservative and did not overstate model quality, offering generalizable guidance for LLM deployment in healthcare AI with emphasis on reproducibility.

Supplementary Note 4: Prompt for Creating Iodinated Contrast Media (ICM) Consultation Scenarios

LLM Prompt Design

Please create role-play scenarios depicting consultations between referring clinicians and radiologists regarding contrast-enhanced CT examinations. Each scenario should include

the referring physician's inquiry and the radiologist's response (limited to 5 sentences or fewer).

Format:

#1 Referring Clinician's Inquiry *[Include the clinical question or concern about contrast administration]*

#2 Radiologist's Response *[Provide a concise response in 5 sentences or fewer]*

#3 Key Educational Points *[After the radiologist's response, include the following points in bullet form:]*

1. **Patient Context**: Specific details about the patient's age, gender, medical history, current symptoms, and examination purpose.

2. **Examination-Related Risks and Concerns**: Consider potential risks such as radiation exposure, contrast media allergies, renal function impact, etc.

3. **Alternative Examination Options**: Discussion of alternatives to contrast-enhanced CT, such as MRI, ultrasound, or non-contrast studies when relevant.

4. **Patient/Family Communication**: Reference to explaining risks and benefits to patients or family members, including the informed consent process.

5. **Post-Examination Follow-up**: Mention any necessary post-examination observation or additional care planning.

Example:

> **Referring Clinician**: "I'd like to request a contrast-enhanced CT scan for a 68-year-old male patient for follow-up of an abdominal aortic aneurysm. His eGFR is 38 mL/min/1.73m². What are your thoughts on the risks versus benefits of using contrast media in this case?"
>
> **Radiologist**: "This patient's eGFR falls within the 30-45 mL/min/1.73m² range, which indicates increased risk for contrast-induced nephropathy (CIN), but iodinated contrast media can still be administered with appropriate precautions. For optimal evaluation of an abdominal aortic aneurysm, a contrast-enhanced CT provides the most valuable diagnostic information. I recommend proceeding with the contrast study while implementing specific risk-mitigation strategies."

Key Points:

1. Optimize contrast type and volume, using low-osmolality contrast media when possible.
2. Ensure adequate hydration before and after the examination, considering intravenous normal saline administration if appropriate.
3. Recommend temporary discontinuation of nephrotoxic medications (such as NSAIDs) prior to the procedure.

4. Plan for reassessment of renal function 48-72 hours post-contrast administration.
5. Alternative imaging modalities (non-contrast CT, ultrasound) were considered but deemed insufficient for detailed aneurysm evaluation in this case.

Supplementary Note 5: Prompt for Iodine Contrast Agent Consultation

LLM Prompt Design

# Role

- You are an experienced radiologist with expertise in CT and contrast examinations.

# Requirements

- Please respond to inquiries from referring physicians about contrast agent usage, addressing safety, dosage adjustments, alternative diagnostic methods, and risk management.
- Please consider the urgency, patient's medical history, and the referring physician's experience level.

# Process Please follow these steps:

- Classify patient risk using a three-tiered system (Low, Moderate, High).
- Evaluate the necessity and feasibility of contrast agents.
- Provide an overview of the patient's condition.
- Categorize the risk with rationale.
- Confirm the question and understand specific clinical concerns.
- Provide evidence-based recommendations.
- Suggest consulting a radiologist for high-risk cases.
- Suggest alternative imaging strategies if applicable.
- Offer opportunities for further consultation if needed.

# Response Format Please compose responses in two paragraphs:

- **Major Paragraph**: A concise answer with key recommendations and the identified risk level.
- **Minor Paragraph**: Supportive information, guideline references, and relevant caveats or disclaimers.

# Rules

- Maintain a professional tone with appropriate medical terminology.
- Adjust technical depth to the referring physician's specialty/experience.
- Prioritize patient safety.
- Use current research and clinical guidelines.
- Acknowledge uncertainties and request additional information when needed.

Supplementary Note 6: LLM-Driven Evaluation Prompt

System

You are an expert medical evaluator specializing in radiology AI responses. Please score each LLM-generated response against a set of predefined metrics.

Input Data

The input provided to the evaluator LLMs included:

- The response time of each evaluated LLM
- The anonymized text response itself
- A reference answer or radiologist-crafted standard

Evaluation Criteria

The following six metrics (with the respective maximum point values) were used:

1. Clinical Accuracy (25 points)

- Appropriateness of Risk Assessment (10 points)
- Adherence to Guidelines (10 points)
- Correctness of Dosage Recommendations (5 points)

2. Response Structure (10 points)

- Logical Flow and Organization (10 points)

3. Safety Considerations (20 points)

- Patient Safety Prioritization (10 points)
- Risk Mitigation Strategies (10 points)

4. Professional Communication (20 points)

- Medical Terminology Usage (10 points)
- Clarity of Explanation (10 points)

5. Practical Applicability (10 points)

- Clinical Relevance (5 points)
- Implementation Feasibility (5 points)

6. Response Time (10 points)

- <1s: 10 points
- 1–2s: 9 points
- 2–5s: 8 points
- 5–8s: 7 points
- 8–10s: 6 points

- 10s: 5 points

Expected Output

Evaluation Table: Tabulated scores per criterion, plus a final overall score for each LLM.

Detailed Evaluation Comments and Overall Conclusion: A brief narrative or bullet-point summary for each criterion and a concluding statement regarding each LLM's performance.

Additional Instructions

Evaluators were instructed to remain fair and unbiased, focusing on each response's clinical correctness and clarity rather than style alone.

Supplementary Figures

Supplementary Figure 1: Model Performance Comparison Across Evaluators. Heat maps showing (A) 1st place achievement rates and (B) mean performance rankings across four different evaluators (one radiologist and three LLM-based judges). Values in panel A represent percentage of cases where each model achieved first place ranking. Values in panel B represent mean ranks where lower numbers indicate better performance.

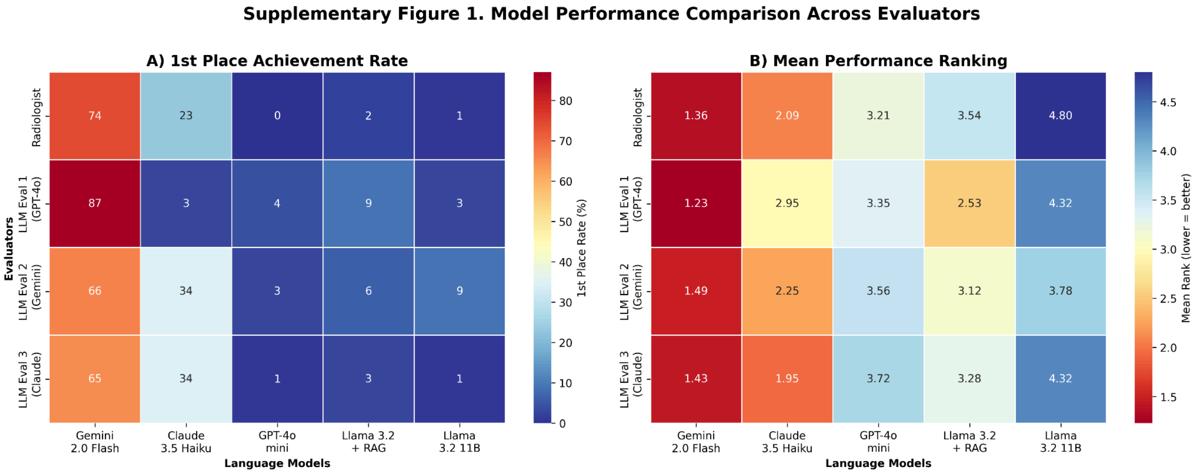

Supplementary Data Files

Supplementary Data 1: Complete ICM Consultation Scenarios Dataset. CSV file containing 100 synthetic iodinated contrast media consultation scenarios used in the main study, including scenario IDs, clinical inquiries, primary categories, and scenario types.

Supplementary Data 2: Temperature Sensitivity Analysis Scenarios. Dataset of 22 additional ICM consultation scenarios specifically developed for temperature parameter validation analysis.
